# Proteoglycan metabolism, cell death and Kashin-Beck Disease

**DOI:** 10.1007/s10719-012-9421-2

**Published:** 2012-06-26

**Authors:** Siyuan Li, Junling Cao, Bruce Caterson, Clare E. Hughes

**Affiliations:** 1 Key Laboratory of Environment and Genes Related to Diseases (Xi’an Jiaotong University), Ministry of Education, Xi’an, China 710061; 2 Connective Tissue Biology Laboratories, Division of Pathophysiology and Repair, School of Biosciences, Cardiff University, Cardiff, UK CF10 3AX; 3 The Institute of Endemic Disease, Medical School of Xi’an Jiaotong University, Xi’an, Shaanxi China 710061

**Keywords:** Kashin-Beck Disease, Proteoglycan, Chondrocyte, Necrosis, Apoptosis, Oxidative stress

## Abstract

Kashin-Beck Disease (KBD) is an endemic, chronic and degenerative osteoarthropathy principally occurring in children. The characteristic pathological change of KBD is chondrocyte necrosis in hyaline articular cartilage. Proteoglycans are one of the major components in the extracellular matrix of articular cartilage, and disrupted proteoglycan metabolism and loss of proteoglycans in articular cartilage from KBD patients has been observed. In this mini-review, we discuss the close relationship between chondrocyte death including necrosis and loss of proteoglycan, and its potential mechanism during KBD onset and development, which may provide new clues for KBD research.

## Introduction

Kashin-Beck Disease (KBD) is an endemic, chronic and degenerative osteoarthropathy affecting approximately 3 million people in China [1, 2]. It principally occurs in childhood [3], with the breakdown of cartilage starting in children as early as 2 or 3-years-old [1], and results in growth disturbances [3]. Its main clinical symptoms include diarthrodal joint deformation and limited joint mobility. In the most severe cases, decreased limb length and short stature are also observed due to the focal and irregular closure of the growth plates [2]. The characteristic pathological change of KBD is chondrocyte necrosis in hyaline cartilage including articular cartilage and growth plates from different peripheral joints. Other pathological changes include cartilage surface fibrillation, chondrocyte cluster formation and more importantly, matrix destruction resulting in the loss of proteoglycans (PGs) [1, 2]

PGs are one of the major components in the extracellular matrix (ECM) of articular cartilage. They are remarkably complex macromolecules consisting of one or several types of polysaccharide glycosaminoglycan (GAG) side chains and a unique core protein. Depending upon the nature of their GAG chains, PGs can be categorised as heparan sulphated PGs (HSPGs), keratan sulphate PGs (KSPGs), chondroitin sulphate PGs (CSPGs) and dermatan sulphate PGs (DSPGs). Alternatively, PGs can also be named according to their unique core proteins, such as aggrecan [4].

Aggrecan is one of the major PGs distributed in the ECM of articular cartilage. It contains both CS/DS and KS GAG side chains attached to an extended core protein. Several aggrecan monomers can bind to hyaluronan, and this non-covalent interaction is stabilised through an additional interaction with link protein so forming stable ternary complexes, especially in the pericellular and territorial matrix around chondrocytes in articular cartilage [5]. Aggrecan plays an important role in the biomechanical properties of cartilage and hence contributes to the main function of cartilage in synovial joints, to withstand compressive loading during movement [5]. The highly negatively charged GAG chains of aggrecan attract the influx of positive ions such as Na^+^ and K^+^, which increases the osmotic pressure in the tissue. This contributes to the large water content present in the ECM of articular cartilage and thereby provides the biomechanical properties to resist the compressive loading applied to joints. Therefore, the function of articular cartilage is dependent on a high aggrecan concentration being present in the tissue ECM [6]. In degenerative joint diseases such as osteoarthritis (OA) and rheumatoid arthritis (RA), loss of PGs especially aggrecan from articular cartilage is regarded as the key initial event in disease onset and development [7] and is considered as a target for intervention that would slow the progression of disease. This is followed by collagen network degradation, which is considered to be an irreversible event for disease development.

**Fig. 1:**
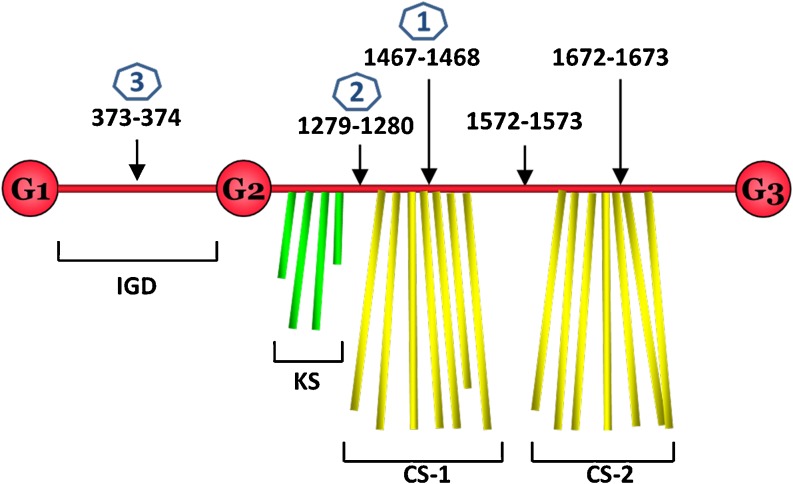
Schematic demonstration of aggrecan degradation. The domain structure of aggrecan core protein with different regions is shown. G1, 2 and 3 are the globular domains, IGD is the interglobular domain, KS, CS-1 and CS-2 are the glycosaminoglycan (keratan sulfate and chondroitin sulfate) attachment regions. Proteolytic cleavage sites on the core protein of aggrecan are displayed by *black arrows*. The blue number in hexagon denotes the preferred order of cleavage

Mechanisms of PG loss in OA and RA have been elucidated and involve increased presence of the inflammatory cytokines interleukin 1 (IL-1) and tumour necrosis factor (TNF) that induce chondrocytes and synovial cells to produce and secrete Matrix MetalloProteinases (MMP) and A Disintegrin And Metalloproteinase with Thrombospondin Motifs (ADAMTS) proteinases that are responsible for the degradation of collagens and PGs, respectively [8]. The pathological sites of cleavage within the core protein of aggrecan by these enzymes is well documented (Fig. 1) and results in the loss of the GAG rich domains of the core protein from the tissue. Although the underlying aetiologies between KBD and the other degenerative joint diseases are different, similar mechanisms of cartilage degradation, *i.e.* PG loss and matrix destruction have been proposed and studied in KBD.

## Proteoglycan catabolism in KBD

Evidence for PG catabolism in KBD patients has been provided from several previous studies in which GAG depletion has been observed in the articular cartilage from KBD patients [2, 9], indicating that the loss of PGs plays a pivotal role during KBD development. Interestingly, a distinct difference in the loss of PGs from the different zones of articular cartilage was seen between juvenile and adult patients [2, 9, 10]. In the juvenile KBD patients, loss of sulphated GAG (sGAG) staining in articular cartilage was mainly localised to areas within the deep zone where chondrocyte necrosis was observed [2, 9]. However, there was sGAG staining in the other zones of articular cartilage, suggesting the association of PG loss with cell death during KBD onset in children. In contrast, the depletion of sGAG was observed in the surface and middle zone of articular cartilage from adult KBD patients, similar to that seen in osteoarthritic patients [2, 10]. Therefore, it is now believed that KBD pathology in adults is more like sub-type of OA with a rare aetiology [11] because their pathological and metabolic changes in articular cartilage are quite similar to those observed in OA patients, although some differences in gene expression profiles have been recently reported [12].

The mechanism of PG depletion from articular cartilage of KBD patients is not clearly understood. However, studies suggest that increased catabolism of aggrecan by ADAMTS4 and/or TS5 in articular cartilage may be involved as an elevated expression of BC-13, a neo-epitope generated by aggrecanases through cleavage of the interglobular domain within the core protein of aggrecan, has been observed in articular cartilage from both juvenile and adult KBD patients [2]. MMPs may also contribute to the aggrecan degradation as increased production of MMP-13, one of the major members of the MMP family responsible for pathological changes in degenerative joint diseases, has been recently reported in the synovial fluid from KBD patients [13].

Moreover, inflammatory cytokines IL-1 and TNF may be involved in PG catabolism in KBD patients as elevated levels of these cytokines have been reported in synovial fluid and serum from patients diagnosed with KBD [2, 14–17]. It is thought that joint tissues respond to these increased levels by up-regulating production and secretion of MMPs and ADAMTS proteinases, eventually leading to the degradation of PG in articular cartilage.

## Alteration of glycosaminoglycan metabolism in KBD

Increased catabolism of PG’s in articular cartilage is postulated to cause an increase of GAG fragment release in body fluids such as urine. Analysis of GAG excretion and their molecular weight in urine has been used to ascertain them as potential biomarkers for OA and RA [18, 19]. Similar monitoring and analysis of GAG’s have been carried out in urine collected from KBD patients. Previous studies have shown that disaccharides composing CS chains, monosaccharides including glucuronic acid and hexosamine were significantly increased in the urine of KBD patients, whereas the molecular weight of CS units in acid mucopolysaccharides were decreased [20–22], suggesting elevated GAG catabolism in the connective tissues from KBD patients. Moreover, there were significantly lower sulphation patterns on disaccharides analysed in urine samples from both juvenile [23] and adult [22] KBD patients when compared with those from the healthy, suggesting a disrupted (altered) GAG sulphation in KBD patients. Interestingly, monkeys administrated with water and grains from KBD areas also showed a similar disruption of GAG metabolism (sulphation) [24, 25], suggesting that environmental factors may influence GAG metabolism.

## Chondrocyte death and loss of proteoglycans in KBD

Chondrocyte death including necrosis [26] and apoptosis [27–30] have been observed in the articular cartilage from KBD patients. Published data indicates that PG loss from juveniles is associated with chondronecrosis but loss and development of degenerative cartilage changes in adult joints may not clearly be associated with the cell death, indicating that cell death may play different roles in PG loss between child and adult during KBD development.

Necrosis is a form of traumatic cell death that results from external factors such as infection, toxins, or trauma. It is accompanied by the early loss of membrane integrity, swelling of cytoplasm, and release of different degradative enzymes including MMPs and ADAMTS proteinases into the adjacent tissue [31]. Therefore, chondrocyte necrosis is highly likely to induce the catabolism of aggrecan and immunohistochemical analysis supports this where specific fragments resulting from cleavage of aggrecan by ADAMTS proteinases was localised around sites of chondrocyte necrosis [2].

Apoptosis is a result of a programmed cell death, which is tightly regulated by cellular signalling events and leads to the orderly disintegration of individual cells with shrinking of cytoplasm, late loss of membrane integrity and no release of degradative enzymes [32]. Therefore, it is unlikely that cell apoptosis will induce the loss of PGs from articular cartilage in KBD patients; this coincides with our and the other’s observation. For example, it has been found that chondrocyte apoptosis is elevated in the superficial and middle zone of articular cartilage from juvenile patients [27, 28], and is not consistent with the areas that PG depletion is seen in the deep zone of articular cartilage from juvenile patients.

However, in the analysis of articular cartilage from adult KBD patients, apoptotic chondrocytes have been observed in the eroded areas where PG depletion is localised [29], indicating that there may exist some linkage between cell apoptosis, PG degradation and progression of the disease. Interestingly, similar observations are seen in osteoarthritic cartilage [29, 33, 34], suggesting that the role of chondrocyte apoptosis in OA and KBD in mature adult cartilage may be occurring through similar mechanistic routes. A potential mechanism has been proposed in which apoptotic cells in cartilage cannot be removed through phagocytosis [35], as chondrocytes are spatially isolated in lacunae and the abundant content of GAGs in the ECM may perturb cell migration. Consequently, chondrocyte apoptosis may either be diverted into secondary necrosis pathways (named aborted apoptosis) [36, 37], or chondroapoptosis, a cell death process involving a combination of apoptosis and autophagy [38, 39]. This process of cell death will eventually induce focal PG degradation in the articular cartilage, and may explain the co-localisation between apoptotic cells and PG depletion in KBD and OA cartilage from adults.

The consequences of cell death in articular cartilage are not only those of PG depletion but also of altered tissue homeostasis. A decrease in chondrocyte number will compromise the tissues ability to repair, and this is an area requiring further research.

## Potential factors altering proteoglycan metabolism in KBD

The precise aetiology inducing chondrocyte cell death and altered PG metabolism in KBD patients is not clear, although several factors have been proposed including selenium deficiency [40], iodine deficiency [41], water pollution with organic material and fulvic acid [42], and mycotoxin contamination in local food [43]. Among these, selenium deficiency and mycotoxin contamination have been extensively investigated and particular attention has been given to the biological effects they have on chondrocyte survival and PG metabolism in articular cartilage.

The first systematic study investigating the effects of different mycotoxins (found in KBD areas) on chondrocyte metabolism was performed by our labs [44]. *In vitro* studies using isolated chondrocytes from articular cartilage and exposure to mycotoxins including deoxynlvalenol (DON), T-2 toxin, nivalenol (NIV), hutenolide (BUT), alternariol methyl ether (AME) and monlliformin (MON) showed increased cell DNA damage, elevated cell mortality and altered PG metabolism. Similar findings have been reported in animal model studies of KBD [45–48]. For example, T-2 toxin and MON have been reported to increase chondrocyte necrosis, reduce sGAG content and sulphation levels in articular cartilage from animals under a selenium deficient nutrition state [45–47]. In a recent study where rats were administrated with T-2 toxin for up to 10 months, loss of PG staining in articular cartilage was reported and involved the entire thickness of the tibial plateaus and femoral condyles [49].

Interestingly, selenium deficiency alone cannot induce significant changes described above [45–47], and the alterations in articular cartilage from these animal models would be much milder if the animals were administrated with mycotoxin under a normal selenium nutrition condition, indicating that the supplement of selenium in the food can alleviate the damages induced by mycotoxins [45–47, 50]. Collectively, these results suggest a combination effect of selenium deficiency and mycotoxins on chondrocyte death and PG catabolism.

In contrast to PGs, mycotoxins did not significantly influence collagen metabolism in articular cartilage from these animal models, although several recent *in vitro* studies have shown some mycotoxin-induced changes in type II collagen at gene and protein levels [51–53]. This indicates that, similar with the other degenerative joint diseases such as OA, in KBD PGs are more vulnerable to loss and damage than the collagen networks, although the causes of their initial stages of degradation may be different.

**Fig. 2:**
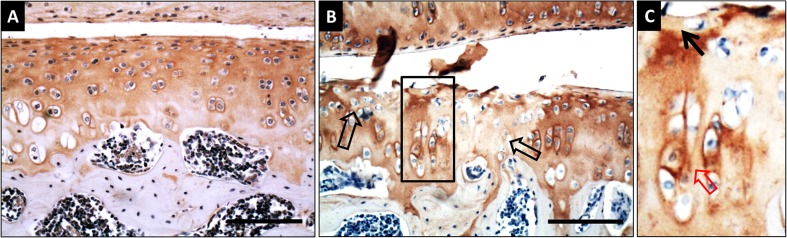
Aggrecan immunohistochemical staining in articular cartilage from rat KBD animal model. KBD rat model was produced by administration with T-2 toxin and selenium deficiency food for 4 weeks. **a** aggrecan positive staining evenly distributes across the whole section of articular cartilage from control group. **b** Focal depletion of aggrecan staining (*hollow black arrows*) was observed in the articular cartilage from T-2 toxin and selenium deficiency group. The area enclosed with back square was magnified and displayed in **c**, note the depletion of aggrecan staining in pericellular matrix of a single chondrocyte (*black arrow*), and the chondrocyte cluster formation and its strong aggrecan staining adjacent to the focal proteoglycan loss area (*hollow red arrow*). Scale Bar: 50 μm

The fact we must emphasize here is that there is no direct evidence between cell death and PG depletion in the articular cartilage in these animal model studies [45–48], because the cell death induced by mycotoxins was mainly demonstrated by histological staining whereas the decreased GAG content in articular cartilage was measured biochemically following extraction. However, a recent rat study in our lab has shown that the depletion of aggrecan induced by T-2 toxin and selenium deficiency was mainly localised to the focal areas where cell death occurred (Fig. 2), indicating a close relationship between PG loss and chondrocyte necrosis induced by mycotoxin. We also investigated chondrocyte apoptosis in the articular cartilage from these experimental rats and found that it was selenium deficiency but not T-2 toxin that significantly induced cell apoptosis; moreover, there was no apparent correlation between the distribution of apoptotic chondrocytes and the localisation of aggrecan depletion (unpublished data). This suggests that selenium deficiency induced cell apoptosis is not directly linked to or a cause of loss of PGs from articular cartilage. However, extensive cartilage damage was observed in experimental rats administered with mycotoxin under a low selenium diet when compared to animals on a low selenium diet or mycotoxin alone. This suggests that selenium deficiency may be an essential “pre-challenge” for chondrocytes before their exposure to the “fatal attack” of mycotoxins, which eventually induces cell necrosis, and consequently the focal PG depletion in articular cartilage.

The mechanism by which these two risk factors work together is not clear. Previous studies have shown that mycotoxins including T-2 toxin mainly target on cell membranes by increasing oxidative stress within cells [54], suggesting that the elevated reactive oxygen species may play a pivotal role. Selenium is a key element for several antioxidant selenoproteins including glutathione peroxidase (GPx) enzymes [55]; its deficiency will dramatically reduce the function of antioxidant systems in cells. On the basis of this “pre-challenge”, the damage induced by mycotoxins could be greatly aggravated, and consequently induce chondrocyte necrosis which results in the focal PG depletion in articular cartilage. This deduction is consistent with our recent *in vivo* study [56], and may explain the synergistic effect of selenium deficiency and mycotoxins described above.

Alternatively, mycotoxins could disrupt PG metabolism directly. It has been reported that T-2 toxin significantly inhibited aggrecan gene and protein expression in chondrocytes cultured *in vitro* [52, 57], but increased the corresponding gene expression of degradative enzymes such as aggrecanase-2 [57]. Moreover, mycotoxins can increase the production of pro-inflammatory cytokines such as IL-1β and TNF-α *in vitro* [58, 59] and *in vivo* [60], similar to their elevated levels reported in KBD patients [2, 14, 16, 17, 61, 62]. These increased levels of pro-inflammatory cytokines could undoubtedly promote the catabolism of PGs in the ECM of articular cartilage. This may explain the significant inhibition of PG synthesis in chondrocytes cultured with serum from KBD patients [63, 64], where elevated TNFα and IL-1β levels were detected.

**Fig. 3:**
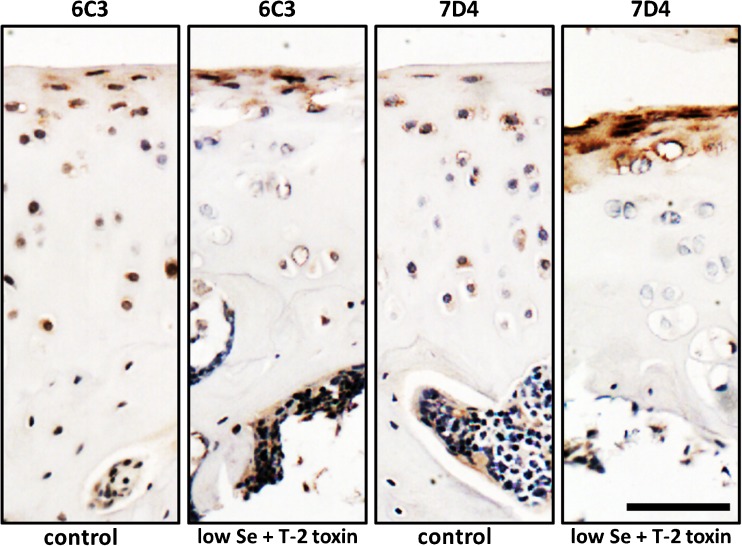
Immunostaining for chondroitin sulphate (CS)/dermatan sulphate (DS) expression in articular cartilage from KBD animal model. KBD rat model was produced by administration with T-2 toxin and selenium deficiency food for 4 weeks. The expression of different epitopes (6C3 and 7D4) along CS/DS chains on proteoglycans in articular cartilage was investigated using immunohistochemical staining (red colour). 6C3 and 7D4 positive staining is mainly localised in the pericellular matrix. In the articular cartilage from control group, 6C3 and 7D4 positive staining is observed in all layers, although the most of the intensive staining is in the superficial and middle zone. In contrast, there is no or very weak 6C3 and 7D4 positive staining in middle and deep zones of articular cartilage from low selenium diet plus T-2 toxin (KBD model group). Interestingly, much stronger positive staining of 6C3 and 7D4 epitopes is observed in the superficial zone of articular cartilage from KBD model group. These results indicate the alteration of CS/DS metabolism induced by selenium deficiency nutrition and T-2 toxin. Scale Bar: 50 μm

Mycotoxins may also interfere with GAG metabolism and subsequently influence PG maintenance and function in articular cartilage. Recently, in our lab an immunohistochemical study using CS/DS motif antibodies has been carried out on articular cartilage taken from rats administered a low selenium diet and/or T2 toxin. This study indicates that there is altered expression of CS/DS GAG chains under the different experimental conditions (Fig. 3), providing evidence that altered GAG synthesis maybe involved in the pathogenesis of KBD.

## Proteoglycan metabolism, chondrocyte death and biomechanical consequences

One unique pathological change in the articular cartilage from KBD patients is the “focal” cell death and its associated “localised” loss of PGs in the ECM. Similar changes have also been observed in different KBD animal models [45–48, 65, 66] including mycotoxins and selenium deficiency induced pig, rat and chicken models. However, it is not clear why this characteristic change is restricted to regional areas of the articular cartilage, although it is most likely related to the different biomechanical distribution across the articular cartilage. Unfortunately, related biomechanical studies have not been performed, especially in the articular cartilage from either KBD patients or animal models. Therefore, the following discussion is largely based upon the knowledge obtained from studies of other degenerative joint diseases such as OA.

There is a distinct compressive loading (from 1 MPa to 25 MPa) applied to the articular cartilage in major joints such as the knee [67], which will greatly influence chondrocyte metabolism and PG turnover [68]. In healthy articular cartilage, physiological compressive stimulation usually increases the synthesis of PGs to help resist the mechanical loading, which is negatively regulated by nitric oxide (NO)-dependent signalling pathways [68, 69]. However, under pathological conditions such as KBD, the elevated oxidative stress [56] and high levels of NO [28] in articular cartilage could severely interfere with the biomechanotransduction signalling cascades and subsequently inhibit PG synthesis in chondrocytes in response to loading [70]. Therefore, chondrocytes may not maintain normal PG production in response to mechanical loading, which could greatly change the mechanical properties of the pericellular matrix; this change could subsequently induce abnormally higher mechanical load applied on cells, further interfering with chondrocyte metabolism. At the end of this process, chondrocytes cannot maintain their microenvironment and thereby cell death is induced. Alternatively, compressive loading could directly induce chondrocyte death but mainly at high strain rate (non-physiological) as reported previously [71, 72]. However, in KBD patients, the threshold of chondrocyte mortality in response to compressive loading is greatly decreased [73] under pathological conditions such as high oxidative stress. Therefore, cell necrosis and subsequent focal PG depletion could happen in the areas of articular cartilage resisting higher (but still physiological) compressive loading. Collectively, these two possibilities may explain the focal cell death and PG depletion in the articular cartilage from both KBD patients and animal models. Of course, biomechanical studies are needed in future studies.

## Therapeutic targets of KBD

Currently, there are no specific treatment methods for KBD patients available due to the unknown aetiologies of this disease. However, based on the selenium deficiency and mycotoxin contamination hypotheses, a series of measures have been performed in KBD endemic areas including provision of the susceptible population (especially children) with dietary grain imported from non-KBD areas and selenium supplements [74], which have greatly decreased the prevalence of KBD [75, 76]. Due to the similar pathological changes between OA and adult KBD patients, several treatments used for OA patients including GAG supplement [77, 78], hyaluronic acid injection into joint space [79] have been tested on KBD patients and these have shown some benefits. Considering the high levels of TNF and IL-1 in synovial fluid from KBD patients and the pivotal role these cytokines play in PG degradation, another potential therapeutic target for KBD are the anti-inflammatory cytokines. Unfortunately, the related clinical trials on KBD patients are still ongoing.

## Conclusion

Although KBD has been known and studied for many years, the precise pathological mechanism of the disease is still poorly understood. It is now believed that KBD is not caused by a single risk factor but by multi-risk factors. The disease is characterised by pathological changes in the articular cartilage by focal chondrocyte death (necrosis) and an associated PG depletion. This may be derived from the risk factor-induced disruption of PG metabolism and subsequent abnormal biomechanical strain distribution applied on chondrocytes. In this mini-review, we propose mechanisms to explain how chronic matrix remodelling processes and cell death contribute to structural damage that characterizes KBD. More research is required to fully characterize the detail mechanism of KBD.
